# Time Lags between Exanthematous Illness Attributed to Zika Virus, Guillain-Barré Syndrome, and Microcephaly, Salvador, Brazil

**DOI:** 10.3201/eid2208.160496

**Published:** 2016-08

**Authors:** Igor A.D. Paploski, Ana Paula P.B. Prates, Cristiane W. Cardoso, Mariana Kikuti, Monaise M. O. Silva, Lance A. Waller, Mitermayer G. Reis, Uriel Kitron, Guilherme S. Ribeiro

**Affiliations:** Centro de Pesquisas Gonçalo Moniz, Salvador, Brazil (I.A.D. Paploski, M. Kikuti, M.M.O. Silva, M.G. Reis, U. Kitron, G.S. Ribeiro);; Universidade Federal da Bahia, Salvador (I.A.D. Paploski, M. Kikuti, M.G. Reis, G.S. Ribeiro);; Secretaria Municipal de Saúde de Salvador, Salvador (A.P.P.B. Prates, C.W. Cardoso);; Emory University, Atlanta, Georgia, USA (L.A. Waller, U. Kitron)

**Keywords:** Zika virus, viruses, Guillain-Barré syndrome, time lags, outbreaks, exanthematous illness, microcephaly, epidemiology, pregnant women, Salvador, Brazil

## Abstract

Zika virus infection emerged as a public health emergency after increasing evidence for its association with neurologic disorders and congenital malformations. In Salvador, Brazil, outbreaks of acute exanthematous illness (AEI) attributed to Zika virus, Guillain-Barré syndrome (GBS), and microcephaly occurred in 2015. We investigated temporal correlations and time lags between these outbreaks to identify a common link between them by using epidemic curves and time series cross-correlations. Number of GBS cases peaked after a lag of 5–9 weeks from the AEI peak. Number of suspected cases of microcephaly peaked after a lag of 30–33 weeks from the AEI peak, which corresponded to time of potential infections of pregnant mothers during the first trimester. These findings support the association of GBS and microcephaly with Zika virus infection and provide evidence for a temporal relationship between timing of arboviral infection of pregnant women during the first trimester and birth outcome.

## Abstract 2

There is strong evidence of a temporal relationship between virus infection in pregnant women and birth outcome.

In late 2014, cases of acute exanthematous illness (AEI), involving widespread rash of unclear etiology, were reported in several municipalities in northeastern Brazil. By April 2015, Zika virus was identified in patients from the states of Bahia (*1*) and Rio Grande do Norte, Brazil (*2*). In Salvador, the capital of Bahia, during February–June 2015, ≈15,000 cases of indeterminate AEI were reported (*3*). Reverse transcription PCR performed on 58 serum samples from AEI outbreak case-patients identified Zika virus in 3 (5.2%) of them. (*3*). Although chikungunya and dengue viruses were also detected at similar frequencies, the low frequency of fever (35.1%) and arthralgia (26.5%) among AEI patients suggested that Zika virus was the likeliest etiology for the outbreak (*3*).

The virus has continued to spread, and by the end of 2015, laboratory-confirmed autochthonous Zika virus cases have been identified in all 5 regions of Brazil; the Brazilian Ministry of Health estimated that 500,000−1.5 million persons were infected (*4*). Zika virus has since spread to other regions of the Americas and resulted in large epidemics (*5*).

Studies conducted during a Yap Island (Federated States of Micronesia) outbreak found that ≈20% of Zika virus infections showed clinical symptoms (*6*). For most patients in whom symptoms develop, the disease is self-limited and clinical manifestations (exanthema [rash], arthralgia, fever, and conjunctivitis) are mild (*6*). However, during the outbreak in French Polynesia, a 20-fold increase in the incidence of GBS was observed (*7*), and concerns about an association between Zika virus infection and GBS were first raised. A case–control study subsequently identified strong associations of GBS with positive Zika virus seroneutralization and Zika virus IgM or IgG (*8*). Since 2015, an increase in GBS rates has also been observed in Brazil, Colombia, El Salvador, Suriname, and Venezuela (*9*).

The increase in newborns with microcephaly in northeastern Brazil in late 2015 called global attention to Zika virus as a major public health threat to pregnant women and their newborns (*10*). Even without a conclusive association between a prenatal Zika virus infection and neurologic disorders in the offspring, the Brazilian Ministry of Health and World Health Organization declared a public health emergency (*11*). Since then, clinical evidence increasingly supports an association of prenatal Zika virus infection with birth of babies with microcephaly, and other neurologic and ophthalmologic complications, as well as miscarriages and stillbirths (*12*–*17*).

Salvador, the largest city in northeastern Brazil (2015 population of 2.9 million persons) has been one of the main epicenters for epidemics of Zika virus infection, GBS, and microcephaly. Using raw and smoothed temporal data collected during these outbreaks, we investigated the temporal associations and determined the time lags between epidemiologic curves of the suspected Zika virus infection outbreak, reported cases of GBS, and reported suspected cases of microcephaly.

## Methods

### Data Collection and Case Definitions

In April 2015, the Centers for Information and Epidemiologic Surveillance of Salvador (CIES) established 10 public emergency health centers as sentinel units for systematic surveillance of patients with AEI of unknown cause in Salvador. A case-patient was defined as a resident of Salvador who had a rash, with or without fever, and whose clinical and epidemiologic characteristics did not satisfy the criteria for dengue, chikungunya, measles, or rubella (*18*). The public health units searched retrospectively for suspected cases by review of medical charts of patients treated starting on February 15, 2015; continued with prospective case detection; and submitted weekly reports of identified cases to CIES. On May 25, 2015, because of the sharp decrease in the number of outbreak cases, CIES reduced the number of sentinel health units to the 3 that reported the most cases, although several of the other units continued to report AEI cases voluntarily. For our analyses, we used the reported number of cases for February 15–December 31, 2015.

After neurologic syndrome cases in adults potentially associated with a previous Zika virus infection were first reported in Salvador in late May, CIES initiated surveillance for hospitalizations caused by neurologic manifestations that might be linked to Zika. Cases were identified retrospectively during April–May and followed by prospective case detection. CIES regularly contacted all city hospital epidemiologic services and investigated all suspected case-patients who resided in Salvador. Surveillance personnel, supported by infectious disease physicians and neurologists, ruled out cases for which clinical and laboratory manifestations indicated other diagnoses, and only included cases of GBS and its variants (e.g., Miller-Fisher syndrome). For our analyses, we used the number of hospitalized patients with GBS or GBS variants identified in Salvador during 2015.

After the increase in number of cases of microcephaly in newborns first noticed in Pernambuco State in September 2015, and the request from the Brazilian Ministry of Health that all suspected cases of microcephaly in newborns be reported, CIES established a reporting system in October 2015. Since then, CIES has requested and received reports of all newborns with suspected neurologic impairments and has been investigating all potential cases of microcephaly.

Suspected cases of microcephaly in newborns were reported on the basis of a reduced occiptofrontal perimeter at birth. The initial criteria for reporting was newborns delivered after >37 gestational weeks with an occiptofrontal perimeter <33 cm, or newborns delivered before 37 gestational weeks with a perimeter less than the third percentile of the Fenton curve (*19*). In December, 2015, the Brazilian Ministry of Health changed the first criterion to an occiptofrontal perimeter <32 cm (*20*).

For our analyses, we only included suspected microcephaly case-patients that fulfilled these latest criteria. The first such case-patient was born on July 11, 2015, and a search of the national information system on live births from Salvador for the AEI outbreak period produced no additional cases of congenital malformation fulfilling these criteria. We included all of suspected cases of microcephaly up to March 10, 2016 (the 10th epidemiologic week of 2016); and data for the last case-patient was updated on March 17, 2016.

We opted to analyze all reported suspected cases of microcephaly, instead of only those investigated and confirmed, because only 27.7% of the reported cases had been investigated. Limiting analysis to only confirmed cases could potentially introduce bias because cases that were reported earlier during the outbreak were more likely to have had the investigation concluded. In contrast, including all reported cases might introduce some false-positive diagnoses. Because both inclusion criteria are not free of a potential bias, we analyzed all reported suspected cases of microcephaly.

CIES served as the repository of all AEI, GBS, and suspected microcephaly data from all contributing sources. CIES evaluated and integrated data, including merging of different reporting spreadsheets, and removed duplicate information (on the basis of name, age, date of reporting, and sanitary districts of residence) and nonsense data (e.g., all missing information). Numbers of cases of AEI, GBS, and suspected microcephaly per epidemiologic week were then tabulated.

### Data Analysis

We analyzed case-patients with AEI, GBS, and suspected microcephaly by date of medical care, date of hospitalization, and date of birth, respectively. We used the documented date of medical care or hospitalization, rather than the presumed day when symptoms began, to avoid recall error and reduce missing information.

We constructed epidemiologic curves by week and with 3-week and 5-week moving averages by using Stata software (*21*). We smoothed data by using 3-week and 5-week moving averages to reduce week-to-week variation, wherein the count of events for a given week was averaged with values of the previous and following weeks (3 weeks) or with the 2 previous and 2 following weeks (5 weeks). Because the weekly increase in cases during the outbreak was much larger than the observed weekly variation, there was little difference between crude and smoothed data.

We assessed temporal correlations between our time series by using standard estimation of lagged time-series cross-correlations (*22*) to identify lag times showing the highest correlations between weekly numbers of AEI and ensuing weekly numbers of 1) GBS cases and 2) suspected cases of microcephaly. Although one could evaluate statistical significance by comparing cross-correlations to those expected under a null hypothesis of no association (*22*), our primary focus was to estimate lags with the strongest correlation (i.e., at what lags do the strongest correlations occur?), not a strict evaluation of whether any correlations occurred. Because both time series showed single large increases, our goal was to identify time lags between these series. Specifically, we examined lag times of 0–40 weeks and compared the AEI time series to those for GBS and suspected microcephaly to cover the full pregnancy period. Because of observed timing of initial epidemic curves, we present only results for positive time lags (i.e., AEI preceding GBS or suspected microcephaly). We also assessed cross-correlations for raw and 3-week and 5-week smoothed data.

## Results

**Figure 1:**
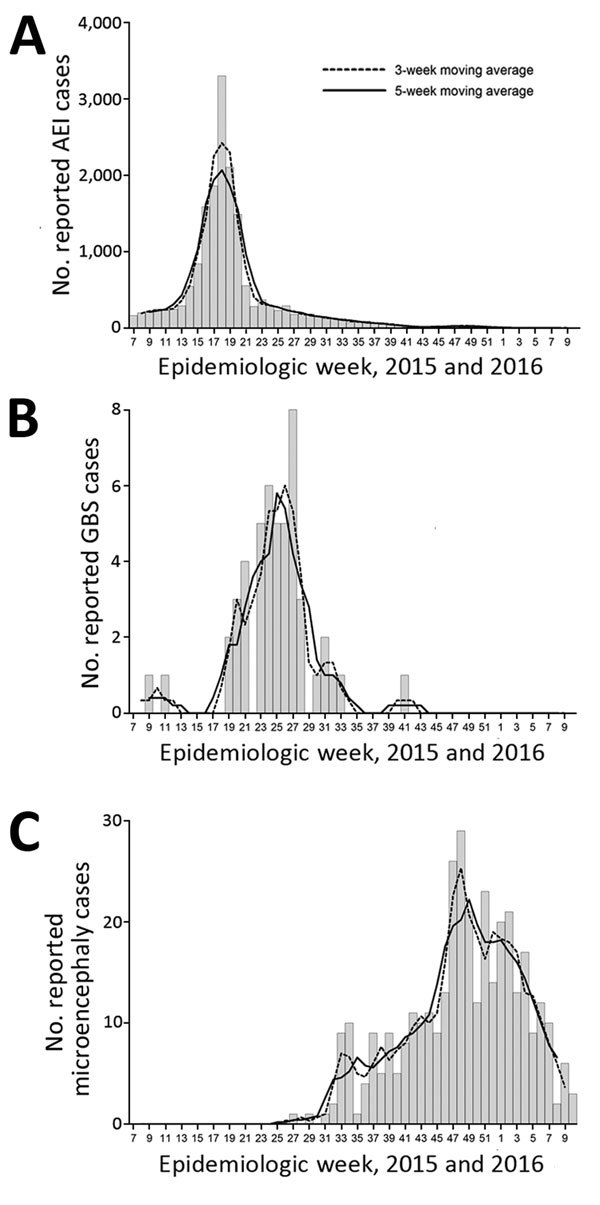
Epidemiologic curves of weekly cases and moving averages of 3 weeks and 5 weeks for A) acute exanthematous illness (AEI), B) Guillain-Barré syndrome, and C) suspected microcephaly, Salvador, Brazil, 2015–2016. The specific starting date during week 7 was February 15, 2105.

During the study, CIES recorded 17,503 reported cases of AEI (5.99 cases/1,000 persons during 2015), 51 hospitalizations of persons with of GBS (1.74 cases/100,000 persons during 2015), and 367 newborns with suspected microcephaly (15.6 cases/1,000 newborns during July 2015–February 2016, which peaked at 31.4 cases/1,000 newborns in December) (Table). Raw and smoothed data (3-week and 5-week moving averages) had a clear initiation, peak, and reduction of cases, and followed a classic epidemic time series of incidence for AEI, GBS, and suspected microcephaly (Figure 1).

**Table:** Cases of reported acute exanthematous illness, Guillain-Barré syndrome, and microcephaly per epidemiologic week, Salvador, Bahia, Brazil, 2015–2016*

Epidemiologic week†	Year	Acute exanthematous illness	Guillain-Barré syndrome	Reported microcephaly
7	2015	161	0	0
8	2015	195	0	0
9	2015	216	1	0
10	2015	245	0	0
11	2015	228	1	0
12	2015	242	0	0
13	2015	288	0	0
14	2015	543	0	0
15	2015	840	0	0
16	2015	1,585	0	0
17	2015	1,861	0	0
18	2015	3,301	0	0
19	2015	2,105	2	0
20	2015	1,486	3	0
21	2015	551	4	0
22	2015	279	0	0
23	2015	366	5	0
24	2015	294	6	0
25	2015	229	5	0
26	2015	289	5	0
27	2015	177	8	1
28	2015	179	3	0
29	2015	181	0	1
30	2015	150	1	0
31	2015	118	2	1
32	2015	127	1	2
33	2015	121	1	9
34	2015	72	0	10
35	2015	77	0	1
36	2015	86	0	4
37	2015	54	0	9
38	2015	50	0	5
39	2015	55	0	9
40	2015	43	0	5
41	2015	12	1	8
42	2015	10	0	11
43	2015	2	0	10
44	2015	14	0	11
45	2015	21	0	9
46	2015	16	0	13
47	2015	17	0	26
48	2015	31	0	29
49	2015	35	0	21
50	2015	4	0	12
51	2015	21	0	23
52	2015/2016	9	0	14
1	2016	NA	0	20
2	2016	NA	0	21
3	2016	NA	0	13
4	2016	NA	0	17
5	2016	NA	0	9
6	2016	NA	0	12
7	2016	NA	0	10
8	2016	NA	0	2
9	2016	NA	0	6
10	2016	NA	0	3

*NA, not available. †The specific starting date during week 7 was February 15, 2015.

Number of AEI cases with available data for date of medical care (16,986 [97.1%]) (Figure 1, panel A) peaked during week 18 (May 3–9, 2015), as reported (*3*). The peak during week 18 was confirmed by 3-week and 5-week moving averages. During weeks 16–20 (April 19–May 23, 2015), >1,000 AEI cases/week were reported.

Number of GBS cases with a known date of hospitalization (49 [96.1%]) (Figure 1, panel B) peaked during weeks 23–27 (June 7–July 11, 2015). Using the 5-week moving average, we found that >4 cases were reported during weeks 23–27. The 5-week and 3-week moving averages provided a clearer picture of the GBS epidemic curve, which was susceptible to higher variability, given the relatively low number of cases per week.

Suspected cases of microcephaly that satisfied our criteria and included a date of birth (357 [97.3%]) (Figure 1, panel C) peaked during weeks 47–49 (November 22–December 12, 2015), during which there were >20 cases/week. Moving averages helped smooth the epidemiologic curve, which is susceptible to uneven time lags between a potential prenatal infection and outcome (i.e., a mother could have been infected at any time during the first trimester or even later). The 18-week period of increase in the number of suspected cases of microcephaly (weeks 31–48) corresponds to a 12-week increase in number of AEI cases (weeks 7–18), and is probably longer because pregnant women throughout the first trimester might have been infected at the onset of the AEI outbreak. For 328 (91.9%) of 357 suspected cases of microcephaly for which data on gestational age at birth were available, the median gestational week was 39 weeks (range 34–41 weeks), which coincided with the first trimester of pregnancy when the AEI outbreak peaked.

**Figure 2:**
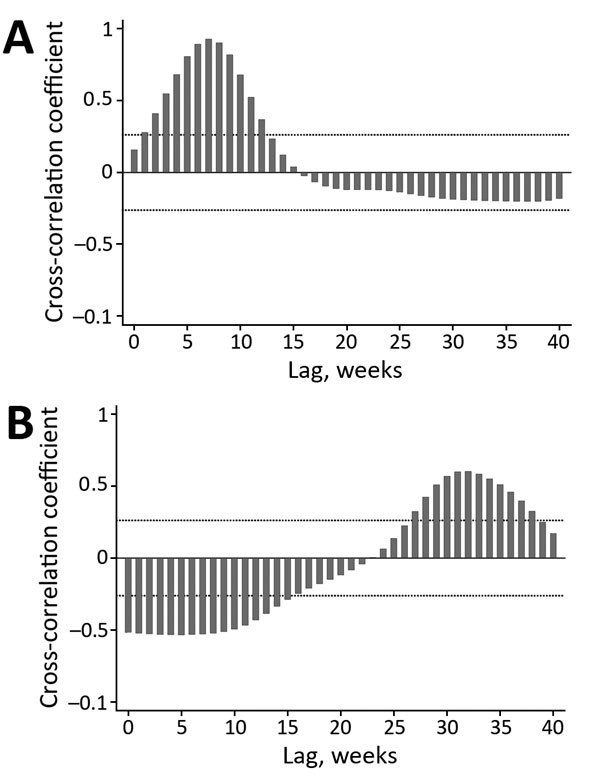
Cross-correlation of acute exanthematous illness with A) Guillain-Barré syndrome and B) suspected microcephaly, Salvador, Brazil, 2015–2016, for a 5-week moving average. Dotted horizontal lines indicate 95% tolerance intervals for a null model of no association. Negative correlations observed at early lag periods are a function of large numbers of acute exanthematous illness cases that occurred early in the study period when there were no suspected cases of microcephaly.

Cross-correlation analyses (Figure 2) confirmed the patterns shown in Figure 1 (i.e., a strong positive correlation between temporally lagged time series driven by observed time lags between peaks in case numbers). Findings were consistent for results based on the raw time series and either the 3-week or 5-week moving averages, and peak correlations differed by <1 week. Number of GBS cases peaked after a lag of 5–9 weeks from the peak in AEI cases (Figure 2, panel A), thus providing strong support for a direct association of the GBS outbreak with the AEI outbreak 1–2 months earlier.

The number of suspected cases of microcephaly peaked after a lag of 30–33 weeks from the peak in AEI cases (Figure 2, panel B), which corresponded to potential infections of mothers during the first trimester of gestation (7–8 months before giving birth). Negative correlations observed at early lag periods were a function of the fact that most AEI cases occurred early in the study period when there were no suspected cases of microcephaly.

## Discussion

Our analyses showed clear and strong cross-correlations for GBS and suspected cases of microcephaly with the original AEI outbreak in Salvador during 2015. These correlations were particularly noteworthy, given delays in case reporting, challenges with diagnosis, and ongoing investigations. Correlations were particularly clear-cut for GBS when a lag of 5–9 weeks from AEI was considered. These results complement a recent case–control study (*8*), which reported an association of GBS with Zika virus in French Polynesia.

Of even more public health interest might be the strong association between outbreaks of AEI and children born with suspected microcephaly (30–33 weeks apart), which demonstrated a strong temporal association between potential exanthematous disease in the first trimester of pregnancy and birth outcome. These results also complement results of studies that linked febrile rash illness suggestive of Zika virus infection during the first trimester of pregnancy and an increased incidence of microcephaly in newborns (*23*,*24*). The ongoing decrease in number of suspected cases of microcephaly in 2016, which has occurred despite continuing and increasing public health and media attention to this serious pregnancy outcome, is particularly noteworthy and matches the reduction in number of cases of microcephaly predicted for Salvador in early 2016 (*25*,*26*).

Recent statements by researchers in Brazil and elsewhere and reports in the media have raised doubts about the actual baseline number of cases of microcephaly in Brazil and questioned the number of cases associated with exposure to Zika virus, given limited baseline data and greatly increased recognition and attention to this phenomenon (*27*). Our results support the link between births of children suspected of having microcephaly and exposure of a pregnant woman to an AEI putatively caused by Zika virus during the first trimester of pregnancy. This link was based on the time-lagged correlation between these 2 factors and the decrease in incidence of congenital manifestations since mid-December 2015.

Although such temporal associations do not prove causation, their strength and pattern makes a major contribution to the growing body of data supporting the association of GBS and congenital malformations with previous exposure to Zika virus (or, at least, an AEI). Furthermore, estimated time lags provide insight into the high-risk exposure period that might lead to these complications and, consequently, help public health and vector control authorities target control and protection efforts more effectively. Additional individual and population level investigations, both clinical and epidemiologic (case–­control and cohort studies) are needed, as are increased resources for surveillance, vector control, and diagnostic capabilities to make definitive connections. With emerging infectious diseases increasing worldwide (*28*), investing in public health surveillance on the city, state, national, and global levels is one of the most cost effective way to help address these ongoing and increasing challenges (*29*).

As an epidemiologic investigation relying on population-level analyses, this study had several limitations. Our data were collected by CIES from different sources, diagnoses were not always definitive, and case definition criteria and case ascertainment were prone to changes, as is common during initial outbreak investigations of novel events. This limitation is particularly true for the AEI outbreak, for which cases were not subjected to an extensive laboratory investigation. In a previous study, we showed that Zika virus, chikungunya virus (CHIKV), and dengue virus were circulating and associated with AEI cases during the outbreak (*3*). On the basis of clinical manifestations for reported AEI case-patients and epidemiologic evidence for the spread of Zika virus in Brazil and to the rest of the Americas, and given the challenges in identifying Zika virus in serum samples, this virus was probably the main arbovirus involved in the AEI outbreak in Salvador during our study. Furthermore, although dengue (*30*,*31*) and chikungunya (*32*,*33*) have been associated with GBS, dengue epidemics have occurred for decades without any associated outbreaks of microcephaly or other severe congenital malformations, and CHIKV infections that occur in pregnant women before the peripartum period do not appear to pose congenital risks (*34*,*35*).

In French Polynesia, during the chikungunya outbreak in 2014–2015, an increase in GBS cases was observed (*33*). Thus, Zika virus and CHIKV might have played a role in emergence of GBS cases in Salvador. Unfortunately, our study design (because of limited available diagnostic data) precluded determining the frequency of each circulating arbovirus during the AEI outbreak. These data are needed to determine whether different arboviral infections peaked at the same time or whether the AEI peak represented the junction of distinct epidemic curves for sequential arbovirus outbreaks.

The presence of 2 infectious triggers, whose temporal distribution might not have coincided at the AEI peak, might partly explain why we observed GBS cases peaking 5–9 weeks after the peak of AEI cases, while in French Polynesia, the lag between peaks of GBS and cases of Zika virus infection was only 3 weeks (*8*). Use of date of medical care for AEI and date of hospitalization for GBS, rather than the presumed day when symptoms began, also might have contributed to the difference in observed time lags. For case-patients for whom data were available, the median interval between AEI symptoms onset and medical care was 1 day, and the median interval between onset of GBS symptoms and hospitalization was 5 days. In addition, patients with AEI might have been less likely to seek medical care for their symptoms, once the community perceived Zika virus infection as benign, making the AEI epidemic curve shorter. Therefore, actual time lags might be shorter than what we observed.

Another limitation was the change in case ascertainment for AEI from retrospective to prospective, and then from using 10 health units to using the 3 units that reported most cases (although several of the other units continued to report AEI cases voluntarily). Retrospective data collection is the common method for detecting a baseline level and initiating an outbreak investigation, and reduction of the number of health units was made after the large decrease in AEI cases. Thus, the effect of these changes on the shape of the epidemic curve is small.

As another limitation, the epidemiologic curve for suspected cases of microcephaly potentially overestimated the actual number of cases. Ongoing investigation of the 5,909 reported suspected cases of microcephaly and other central nervous system impairments in newborns, stillbirths, and abortions in Brazil was completed for 1,687 cases by mid-February 2016. Of these cases, 641 (38.0%) were confirmed (*36*). In Salvador, CIES investigated 99 reported cases of Zika virus congenital syndrome, of which 43 (43.4%) were confirmed.

On the basis of the reported number of suspected cases of microcephaly and the number of births in Salvador during the study, 3.1% of newborns were reported as having suspected cases of microcephaly during the peak month of December 2015. However, if we consider that in December only 20 (58%) of the 34 investigated cases were confirmed, a more realistic estimation for the suspected microcephaly risk in that period is 1.8 cases/100 newborns. We believe that the temporal distribution of reported cases parallels that of actual cases. Also, by analyzing all reported cases, we reduced a major source of observation bias (i.e., investigations of cases reported earlier were more likely to have been completed). The consistent shape and mode of the epidemiologic curves, with or without smoothing, support the robustness of our data and findings.

Our case ascertainments of suspected cases of microcephaly were also potentially influenced by spontaneous and nonspontaneous abortions. Although spontaneous abortions could have occurred because of virus effects during embryogenesis, nonspontaneous abortions might have increased after intense media coverage of the microcephaly outbreak. Abortion is prohibited in Brazil (except for a few situations, such as rape, anencephaly, or risk for death of the mother), but it is commonly performed illegally, and 16.4% of women reported having had >1 abortion (*37*). Unfortunately, no official data are available to help understand the likely effect of abortions on the outbreak of congenital Zika virus syndrome. In addition, the database for suspected microcephaly is restricted to live births, and data on stillbirths and abortions are not available.

Finally, we focused on cross-correlation between the time series because we did not have individual links between GBS cases and earlier AEI in the same person or between suspected microcephaly and prior AEI of the mother. Retrospective studies indicate a recall of AEI by women who have given birth to microcephalic babies, but there are few direct demonstrations of virus transfer (*17*). Use of aggregate data enabled us to test for a temporal association between AEI, GBS, and suspected microcephaly, taking advantage of the establishment in Salvador of a surveillance system for detecting and recording AEI cases early during the outbreak. Consequently, Salvador recorded 17,503 of the 72,062 suspected cases of Zika virus infection in Brazil by February 25, 2016 (*38*).

After the AEI outbreak in Salvador, attention was given to the increased number of cases of microcephaly. However, it is becoming clear that newborns also manifest other congenital malformations (*12*,*16*), and that microcephaly might be the most extreme outcome of arboviral infection of the mother. The recently proposed term congenital Zika syndrome (*39*) might better capture the spectrum of possible clinical manifestation of newborns exposed to Zika virus during gestation. The Brazilian Ministry of Health is now conducting surveillance of microcephaly or changes in the central nervous system (*36*). As neonatal outcomes are likely to be observed in other countries, attention must be given to the full range of potential congenital malformations.
